# A case of generalized argyria presenting with muscle weakness

**DOI:** 10.1186/s40557-017-0201-0

**Published:** 2017-10-02

**Authors:** Inha Jung, Eun-Jeong Joo, Byung seong Suh, Cheol-Bae Ham, Ji-Min Han, You-Gyung Kim, Joon-Sup Yeom, Ju-Yeon Choi, Ji-Hye Park

**Affiliations:** 1 Department of Internal Medicine, Kangbuk Samsung Hospital, Sungkyunkwan University, School of Medicine, 29 Saemunan-ro, Jongno-gu, Seoul, 03181 South Korea; 2 Department of Occupational and Environmental Medicine, Kangbuk Samsung Hospital, Sungkyunkwan University School of Medicine, Seoul, South Korea; 3 Department of Dermatology, Kangbuk Samsung Hospital, Sungkyunkwan University School of Medicine, Seoul, South Korea; 4 Department of Dermatology, Samsung Medical Center, Sungkyunkwan University School of Medicine, Seoul, South Korea

**Keywords:** Generalized argyria, Silver, Myopathy, Polyneuropathy

## Abstract

**Background:**

Argyria is a rare irreversible cutaneous pigmentation disorder caused by prolonged exposure to silver. Herein, we report a case of generalized argyria that developed after chronic ingestion of soluble silver-nano particles and presented with muscle weakness.

**Case presentation:**

A 74-year-old woman visited our emergency room, complaining of fever and mental deterioration. She was diagnosed with acute pyelonephritis and recovered after antibiotic therapy. At presentation, diffuse slate gray-bluish pigmented patches were noticed on her face and nails. Two months prior to visiting our hospital, she was diagnosed with inflammatory myopathy and given steroid therapy at another hospital. We performed a nerve conduction study that revealed polyneuropathy. In skin biopsies from pigmented areas of the forehead and nose, the histopathologic results showed brown-black granules in basement membranes of sweat gland epithelia, which are diagnostic findings of argyria. We reviewed pathology slides obtained from the left thigh muscles and found markedly degenerated myofibers with disorganization of myofibrils without inflammatory reactions, consistent with unspecified myopathy, rather than inflammatory myopathy. The patient was diagnosed with generalized argyria with polyneuropathy and myopathy and transferred to a rehabilitation institution after being tapered off of steroids.

**Conclusions:**

Clinicians should be aware of clinical manifestations of argyria and consider it in differential diagnosis when they examine patients who present with skin pigmentation and muscle weakness.

## Background

Argyria is a rare irreversible cutaneous pigmentation disorder caused by prolonged exposure to silver. The affected area becomes bluish-gray or ash-gray, particularly after sun-exposure [1]. Occupational or iatrogenic silver overexposure is now very rare, but cases of argyria related to the ingestion of silver compounds as folk remedies are still present. Argyria is thought to be harmless and has a benign course, but there have been a few cases of argyria associated with neurotoxicity and myopathy [2, 3]. This is the first case of generalized argyria presenting as polyneuropathy and myopathy in a 74-year-old female following chronic ingestion of soluble silver-nano particles (NPs) as an alternative medicine.

## Case presentation

A 74-year-old female visited our emergency room, complaining of fever and mental deterioration. Her initial vital signs were as follows: pulse rate 111/min, blood pressure 100/60 mmHg, and body temperature, 38.9 °C. On physical examination, no abnormal findings were detected except for left costovertebral angle tenderness. On initial laboratory tests, she had thrombocytopenia (platelet counts, 62 K/mm3). There were no findings of leukocytosis or prothrombin time prolongation. Serum levels of blood urea nitrogen and creatinine were 38.6 mg/dL and 0.6 mg/dL, respectively. The results of liver function tests were within normal limits. The level of C-reactive protein was elevated to 19.24 mg/dL. *E. coli* grew in urine and blood cultures. She was finally diagnosed with acute pyelonephritis and treated with intravenous administrations of ceftriaxone 2 g once daily for 4 days, and then ciprofloxacin 400 mg twice daily for 10 days. She recovered after systemic antibiotics treatment.

At presentation, diffuse and slate gray-bluish pigmented patches were noticed on her face and nails. Neurologic examination showed that the patient’s motor powers of the upper and lower extremities were grade IV and III, respectively. Five years before presentation, the patient had ingested soluble silver-NPs for a year for the purpose of health promotion. She used a colloidal silver generator, which produces 5 ppm of colloidal silver water in a single cycle by electrolysis using silver electrodes. She gave a detailed account to have ingested 0.8-1.2 l of colloidal silver solution daily for approximately 12 months.

Her facial discoloration was first noticed 2 years before presentation. She first experienced muscle weakness of both upper and lower extremities 1 year before presentation. As her muscle weakness gradually worsened, she visited a different hospital seeking a diagnosis 2 months prior to visiting our hospital. She was diagnosed to have inflammatory myopathy after pathological tests of samples obtained from her left thigh muscle at the other hospital, and received steroid therapy. Despite taking oral prednisolone 1 mg per kg body weight per day, she did not show any clinical improvement.

Upon admission to our hospital, serology testing for connective tissue disease-associated autoantibodies including dermatomyositis was performed and there were no abnormal findings for anti-Jo1, anti-Ro/La, or the fluorescent antinuclear antibody, anti-ds DNA, anti-CCP Ab, anti-U1RNP, anti-Sm, or anti-neutrophil cytoplasmic antibody. We performed chest and abdomen computed tomography (CT) to exclude cancer-associated myopathy and did not find any other tumorous lesions. We also performed endoscopic gastro-duodenoscopy and colonoscopy, but observed no discoloration on the gastrointestinal mucosa and no silver particles in biopsy specimens.

**Fig. 1:**
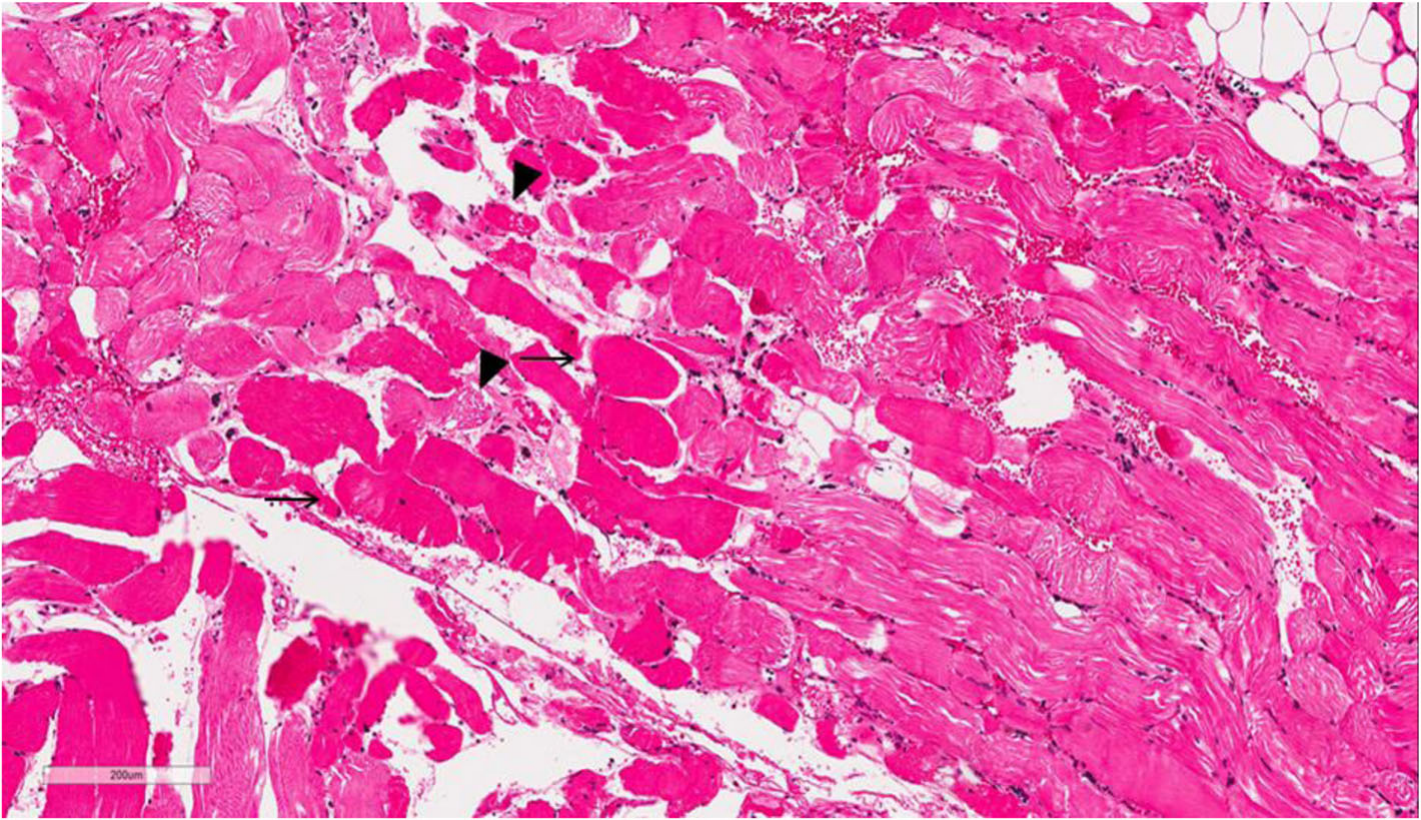
Muscle biopsy (thigh). Myofibers are swollen and hypereosinophilic (*arrow*) and some myofibers are fragmented (*arrowheads*)

**Fig. 2:**
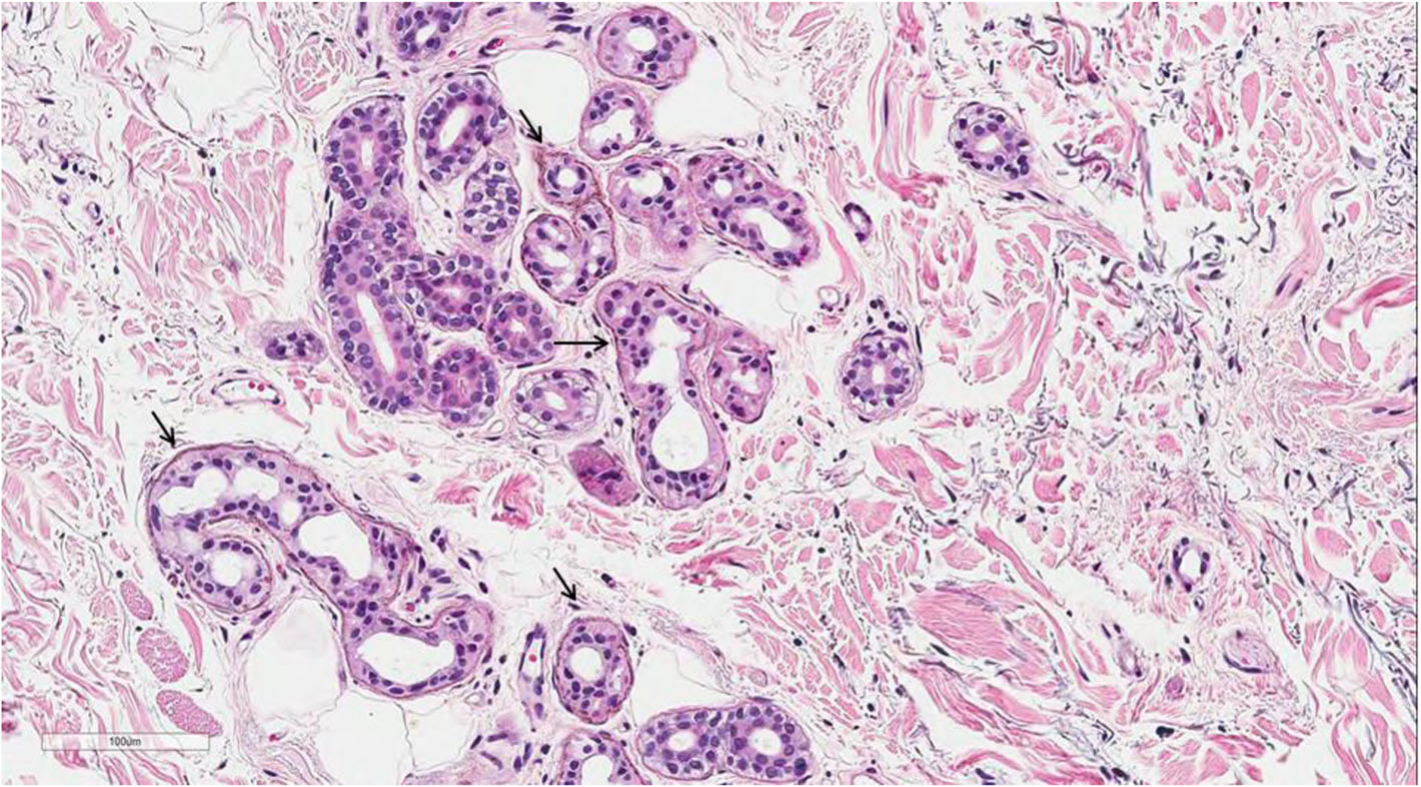
*Brown* to *black* granules lining the basement membrane of sweat gland epithelium (*arrow*, H&E, X200)

Ocular argyrosis was not found in the cornea or conjunctiva. The histopathologic results of skin biopsies from pigmented areas of forehead and nose revealed brown-black granules in the basement membrane of sweat gland epithelium that measured less than 1.0 um in diameter, which are diagnostic findings of argyria (Fig. 1). We reviewed outside pathology slides obtained from the left thigh muscles and found markedly degenerated myofibers with disorganization of myofibrils without inflammatory reactions, consistent with unspecified myopathy, rather than inflammatory myopathy (Fig. 2). We performed electromyography and nerve conduction studies for muscle weakness, which revealed diffuse, mild motor dominant polyneuropathy of both upper and lower extremities. A heavy metal analysis of hair samples including arsenic, mercury, cadmium, and bismuth showed all levels within acceptable ranges and copper levels below normal. We diagnosed the patient with generalized argyria with myopathy and polyneuropathy. She was transferred to a rehabilitation institution after slowly tapering off of steroids on the 22nd day of hospital admission.

## Discussion

Humans have been exposed to silver and its compounds for centuries via the natural environment, industry, and through the use of silver-containing medications. Silver has been used in medications since ancient times. The first mention of silver poisoning in the literature with skin discoloration was reported in 1791, describing a German priest who had used silver nitrate to treat his jaundice [1]. Most cases of argyria in the mid-nineteenth century were related to occupational exposure, in subjects such as silver workers, silver miners, and photographers [2, 3]. In the late 19th and 20th centuries, silver was widely used as cure-all medicine for various conditions such as infectious disease, epilepsy, AIDS, cancer, and arthritis, and therefore cases of argyria associated with silver containing medicines were frequently reported [4]. Since the Food and Drug Administration announced in 1999 that “all over-the-counter drug products containing colloid silver ingredients or silver salts are not generally recognized as safe and effective”, reports of medical overexposures are decreasing. However, there are still a few cases caused by the ingestion of colloidal silver as alternative medicine. In the present case, our patient took colloidal silver approximately 1 year and presented with muscle weakness. She had been misdiagnosed with inflammatory myopathy, resulting in the administration of immunosuppressive agents and subsequent development of APN.

The mechanism and pathogenesis of argyria is not fully understood. Based on animal and human experiments, up to 10% of ingested silver is absorbed in the small intestine, and 2-4% is retained in tissue [5]. After ingestion, the highest concentrations of silver are found in the skin, cornea, and mucosa. Concentrations of silver in liver and kidney are high, as silver is eliminated from the body by those organs. After silver accumulates in tissue as a silver-protein compound, light acts as catalyst by triggering photo-reduction of these compounds to form metallic silver [6]. After metallic silver is oxidized by tissue, it binds as silver selenide and silver sulfide, which are chemically stable and have low solubility. Therefore, pigmentation due to accumulation of silver compounds is permanent. Separately, these compounds promote melanin production, particularly in sun-exposed areas [7]. In our patient, the accumulation of silver was observed in sun-exposed skin by histopathologic examination. To verify argyria-associated myopathy, we reviewed outside pathology slides obtained from left thigh muscles, but silver-containing particles were not found in these slides. Although parenterally-administered silver salts can accumulate in muscles [8], neurons, and glial cells [9], the highest concentrations of silver are usually found in the skin, liver and spleen, with lesser deposits in the muscle and brain [8]. Therefore, the histopathologic diagnosis of silver-deposited myositis and neuropathy is difficult.

Recent commercial application in the forms of NPs, which the same exposure type of silver in our case, is reported to increase the risk for systematic neurotoxicity [10]. Although NPs, defined as one dimension below 100 nm, cannot penetrate a gap of 4-6 nm of the tight junctions in blood brain barrier (BBB), it is likely that NPs influence the endothelial cell damage and permeability of the BBB leading to disruption of tight junctions [11]. NPs can also change the action potential and degenerate the cytoskeletal components and cortical neuron synapse in animal study [12], which may explain the underling mechanisms of polyneuropathy and myopathy in our case. The required dosage of ingested silver to induce polyneuropathy and myopathy is unknown. In this case, it is estimated that the patient had ingested a daily dosage of 4-6 mg of silver NPs, approximately 15-22 times higher than daily oral reference dosage (RfD) of 0.27 mg (0.005 mg/kg/day × 54 kg) derived from United States Environmental Protection Agency (EPA). Lack of data such as silver concentration in serum, neuron and muscles hinders to explain the causal relationship between silver ingestion and neurotoxocity in our case, but this has a clinical significance to show that prolonged ingestion of soluble silver in the forms of NPs may be associated with subsequent development of irreversible myopathy and polyneuropathy in a human case of generalized argyria.

The clinical courses of generalized argyria may depend on the route, extent and types of silver exposure. One case of polyneuropathy associated with topical silver sulphadiazine also was demonstrated with the evidence of both axonal loss and demyelination [13]. The other case of argyric neuropathy we found in the literature may have been caused by silver-based cement used in hip prostheses [14]. However, these cases, unlike our patient, showed reversible courses of neuropathy, which gradually disappeared after cessation of silver. Reversible courses observed in prior cases were related to topical and local application of silver into silver into the skin and joint [13, 14]. The dermal and intraarticular silver exposure might have resulted in a high sliver ion concentration to the adjacent tissues; this usually brings about the local immunologic response rather than systematic effects on neurotoxicity [10].

The diagnosis of argyria is made by histopathological examinations showing typical black silver granules in the basement membrane zone, singly or in clusters, surrounded by sweat glands and connective tissue sheaths around the pilosebaceous structures [15]. Examinations of unstained biopsy sections by dark field illumination show silver granules outlining the basement membranes. Differential diagnoses for blue-gray discoloration include intoxication with other heavy metals besides silver, such as mercury bismuth, arsenic, and gold. In our case, there were no specific systematic diseases or other drug history related to skin pigmentation. All toxic metal levels including arsenic, mercury, cadmium and bismuth were within the acceptable range, except for copper which was lower than the normal range. This effect may be explained because silver alters copper metabolism. In experimental animal models and in humans [16, 17], it has been reported that serum copper concentrations decrease as a result of altered copper metabolism after silver administration. The reduction in cellular copper status observed in our case may therefore be due to silver toxicity.

The present study has some limitations. Polyneuropathy or myopathy has not been observed in previous patients who drank the same colloidal silver solution, but only in two patients with other route of absorption of silver. In addition, the present case showed polyneuropathy or myopathy 3 years after cessation of silver ingestion. Further study is needed to confirm the causality of polyneuropathy with oral intake of colloidal silver solution.

## Conclusions

All silver containing products should be labeled with clear warnings to prevent argyria, especially in alternative health practices. More public education is needed to increase awareness of the neurotoxic effects of NPs in silver-containing products. Clinicians should also be aware of clinical manifestations of argyria and consider it in differential diagnosis when they examine patients who present with skin pigmentation and muscle weakness.

## Abbreviations

AIDS: Acquired immune deficiency syndrome
APN: Acute pyelonephritis
CT: Computed tomography
